# The Synergistic Responses of Different Photoprotective Pathways in Dwarf Bamboo (*Fargesia rufa*) to Drought and Subsequent Rewatering

**DOI:** 10.3389/fpls.2017.00489

**Published:** 2017-04-04

**Authors:** Chenggang Liu, Yanjie Wang, Kaiwen Pan, Qingwei Wang, Jin Liang, Yanqiang Jin, Akash Tariq

**Affiliations:** ^1^Key Laboratory of Mountain Ecological Restoration and Bioresource Utilization & Ecological Restoration Biodiversity Conservation Key Laboratory of Sichuan Province, Chengdu Institute of Biology, Chinese Academy of SciencesChengdu, China; ^2^Key Laboratory of Tropical Plant Resources and Sustainable Use, Xishuangbanna Tropical Botanical Garden, Chinese Academy of SciencesMenglun, China; ^3^College of Life Science, Sichuan Normal UniversityChengdu, China; ^4^Graduate School of Life Sciences, Tohoku UniversitySendai, Japan

**Keywords:** CO_2_ assimilation, energy partitioning, thermal dissipation, the water–water cycle, antioxidative defense system, rewatering

## Abstract

Dwarf bamboo-dominated forests are often subjected to temporary periods of drought due to rising air temperature and decreasing rainfall. Nevertheless, the relationship among CO_2_ assimilation, photoprotective pathways and metabolism of reactive oxygen species (ROS) remains unexplored in bamboo species. Changes in leaf gas exchange, chlorophyll fluorescence, energy partitioning, antioxidative system and compounds related to ROS metabolism in *Fargesia rufa* plants subjected to drought and subsequent rewatering were analyzed. Drought resulted in a reversible inhibition of photochemistry, particularly net CO_2_ assimilation, and lipid peroxidation due to ROS accumulation. Meanwhile, photoprotective pathways, including the water–water cycle (especially for moderate drought), and adjustment in antenna pigments, thermal dissipation and antioxidative defense capacity at organelle levels (especially for severe drought), were up-regulated at the stress phase. Conversely, photorespiration was down-regulated after drought stress. As a result, rewatering restored most of the photochemical activity under drought, especially moderate drought. Moreover, thermal dissipation under severe drought was still operated for avoiding high ROS levels after rewatering. Therefore, the synergistic function of these photoprotective pathways except photorespiration can protect the photosynthetic apparatus from oxidative damage in response to varying intensities of drought stress when CO_2_ assimilation is restricted. This is helpful for the gradual recovery of photosynthetic capacity after rewatering. Thus, *F*. *rufa* plants can withstand drought and is capable of survival in such environment.

**Highlights:**

1. The effects of drought and subsequent rewatering on *Fargesia rufa* were studied.
2. Drought resulted in a reversible inhibition of photochemistry.
3. Photoprotective pathways except photorespiration were up-regulated at the drought phase.
4. Rewatering rapidly restored photochemical activity, especially under moderate drought.
5. *Fargesia rufa* plant is capable of resisting and surviving drought environment.

## Introduction

Climate change is predicted to induce an increase in the severity and duration of drought events in many regions. Drought often limits plant growth and productivity worldwide and continues to threaten the world’s food security. Plants acclimate to drought by regulating physiological and biochemical characteristics wherein photosynthesis is a primary target (Reddy et al., 2004). Usually, drought inhibits photosynthetic CO_2_ assimilation through stomatal (e.g., closure of stomatal and decline of mesophyll conductance) or non-stomatal (e.g., metabolic impairment) factors (Cornic and Fresneau, 2002; Flexas et al., 2004). Inhibition in CO_2_ assimilation will cause excess excitation energy and electron fluxes to O_2_, thus leading to photooxidative damage of the cell components by overproduction of reactive oxygen species (ROS) and ultimately photoinhibition (Parvaiz and Satyawati, 2008).

Plants have evolved multiple protective mechanisms that are thought to cooperate in protecting the photosynthetic apparatus from the potential damage and photoinhibition. For instance, adjustments in light-harvesting antenna size can reduce light energy absorption when the capacity of CO_2_ assimilation declines (Niyogi, 1999). Moreover, non-photochemical (i.e., xanthophyll cycle-mediated thermal dissipation) and photochemical (i.e., photorespiration and the water–water cycle) pathways help to remove excess excitation energy from the photosynthetic electron transport chain (Badger and Takahashi, 2011). Meanwhile, a series of enzymatic and non-enzymatic antioxidants are also involved in coping with excessive energy and detoxifying ROS at the cellular and whole-organism levels (Hu et al., 2005; Silva et al., 2010). Since ROS production may occur in diverse subcellular compartments, including chloroplasts, mitochondria, peroxisomes and cytoplasm, isolated organelles are often used to study their antioxidative response to different stresses (Hu et al., 2005; Song et al., 2009). Although these protective pathways have been well-studied separately, their respective efficiencies under drought conditions vary among plant species and may depend on exposure time to drought (Galmés et al., 2007b; Uzilday et al., 2012). Hence, their synergistic function in mechanism of drought tolerance is actually not understood due to few systematic works.

The impact of drought on photoprotective pathways has been studied in different types of plants, including woody plants (shrubs and trees) and herbs (Guan et al., 2004; Gallé et al., 2007; Zhou et al., 2007; Silva et al., 2015). However, not much information is available in semi-woody plants. Moreover, the capacity of recovery and the involved processes during recovery from drought in plants remain unclear (Flexas et al., 2006; Gallé et al., 2007). Some drought recovery reports showed that the capacity of recovery is associated with the drought tolerance of plants (Souza et al., 2004; Galmés et al., 2007b; Sapeta et al., 2013; Silva et al., 2015). Therefore, studies that would elucidate how plants recover after drought relief will be the right step in ensuring plant survival and growth under drought conditions.

Bamboo plants are a special kind of semi-woody plants. Among them dwarf bamboos, belonging to Bambusoideae, are rhizomatous, perennial and evergreen species. They predominate the main synusia in the understory of several montane and subalpine forests in East and Southeast Asia and South America, and play a major role in preventing soil erosion and increasing forest carbon sequestration (Tsuyama et al., 2012). Dwarf bamboo-dominated forests are often subject to extreme temperature and rainfall patterns as a result of climate change, which exposes them to temporary periods of drought during their life cycle and then adversely affects their growth (Wang and Ma, 1993). *Fargesia rufa* Yi, one of the most important dwarf bamboos, is distributed abundantly in floors of subalpine forests of China (Li et al., 2013). More importantly, it is the staple food for the endangered giant pandas. *F*. *rufa* is extremely sensitive to drought because of its shallow roots with requirement of higher water tables (Wang and Ma, 1993). Drought has been shown to unfavorably affect growth, CO_2_ fixation, and nitrogen metabolism of *F*. *rufa* (Liu et al., 2015a,b), but it is seldom known how the above photoprotective pathways in bamboo plants respond to drought and subsequent rewatering.

Therefore, the present study was conducted to test the hypothesis that dwarf bamboo can employ different photoprotective pathways to cooperatively protect the photosynthetic apparatus against oxidative damage under varying intensities of drought stress, and then recover as soon as possible from drought after rewatering. To verify this hypothesis, the changes in leaf gas exchange, chlorophyll fluorescence, energy partitioning, antioxidative system, and compounds related to ROS metabolism in *F*. *rufa* plants subjected to drought and subsequent rewatering were examined.

## Materials and Methods

### Plant Material and Treatments

The experiment was carried out at Maoxian Mountain Ecosystem Research Station (103°53′ E, 31°41′ N; 1820 m asl), Chinese Academy of Sciences in southwestern China. In March 2013, the healthy and uniform plants (2-year-old, height 40 ± 5 cm) of dwarf bamboo (*F. rufa*) were selected from the nursery at Wanglang National Nature Reserve, and then transplanted into 50 L pots filled with 25 kg homogenized topsoil from the experimental site. Each pot had one standard plant having 4–5 ramets. Thereafter, all plants were placed in a semi-controlled greenhouse with a day/night temperature range of 15–33 and 10–15°C and relative humidity of 50–85%, and watered regularly with water from a nearby stream. Four months after the transplanting, the drought treatments were initiated. The pots were divided into three groups for drought treatments. One group was kept well-watered (WW) as the control [80% relative soil water content (RSWC)] during the whole experiment, and the other two groups were subjected to moderate drought (MD, 50% RSWC) and severe drought (SD, 30% RSWC) respectively for 30 days. The RSWC of each treatment was controlled using the weight method (Xiao et al., 2009; Xu et al., 2009). During the experiment, pots were weighed every other day and rewatered to their respective target RSWC by replacing the amount of transpired water. Evaporation from the soil surface was prevented by enclosing the soil with plastic bags which were tied at the base of each plant. Thereafter, the drought treatments were watered regularly as control for 15 days for recovery. In each treatment, four replications, each including five pots, were used for our experiments. The youngest fully expanded leaves at the same developmental stage were used to analysis various physiological and biochemical parameters.

### Leaf Relative Water Content

Leaf relative water content was determined as described by Galmés et al. (2007a) and calculated according to the equation: LRWC = [(FW – DW)/(TW – DW)] × 100, where FW is leaf fresh weight; DW is leaf dry weight after drying at 70°C for 48 h, and TW is turgid leaf weight after soaking in deionized water for 12 h at room temperature.

### Gas Exchange, Chlorophyll Fluorescence, and Energy Dissipation Analyses

Gas exchange and chlorophyll fluorescence were simultaneously performed using a Li-6400 portable photosynthesis system equipped with a 6400-40 fluorometer chamber (LI-Cor, Inc., Lincoln, NE, USA). The net CO_2_ assimilation rate (*P*_n_), stomatal conductance (*G*_s_), intercellular CO_2_ concentration (*C*_i_), light-adapted maximum (*F*_m_*′*), minimum (*F*_o_*′*) and steady-state fluorescence yield (*F*_s_) were measured between 9:00 and 11:00 am. Environmental conditions in the chamber used for leaf measurements consisted in a photosynthetic photon flux density (PPFD) of 800 μmol m^-2^ s^-1^, a vapor pressure deficit of 1.0–1.5 kPa, a flow rate of 500 μmol s^-1^, an air temperature of 25°C and ambient CO_2_ concentration of 380 ± 5 μmol mol^-1^, respectively. A quantum yield of PSII reaction center photochemistry (Φ_PSII_) and photochemical quenching (*q*_p_) were calculated as (*F*_m_*′* – *F*_s_)/*F*_m_′ and (*F*_m_′ – *F*_s_)/(*F*_m_*′* – *F*_o_*′*), respectively (Silva et al., 2015). After the measurements of the light-adapted parameters, the leaves were darkened using leaf-clips for 40 min. Then a saturation pulse of 8000 μmol m^-2^ s^-1^ was applied for 0.8 s, the maximal fluorescence yields (*F*_m_) and the intrinsic quantum efficiency of photosystem II (PSII) photochemistry (*F*_v_/*F*_m_) was recorded. Non-photochemical quenching was calculated as *F*_m_/*F*_m_*′* – 1. Basing on Φ_PSII_ + Φ_NPQ_ + Φ_f,D_ = 1, the quantum efficiency of photochemical energy dissipation (Φ_PSII_ = 1 -*F_s_*/*F_m_′*), ΔpH- and xanthophyll-mediated thermal dissipation (Φ_NPQ_ = *F_s_*/*F_m_′*-*F_s_*/*F_m_*) and fluorescence and constitutive thermal dissipation (Φ_f,D_ = *F_s_*/*F_m_*) were calculated according to Hendrickson et al. (2004). The flux of energy dissipation via each process (*J*_PSII_, *J*_NPQ_, and *J*_f,D_) was calculated by multiplying the respective quantum efficiency with irradiance and leaf absorption coefficient (α), respectively (Harley et al., 1992; Hendrickson et al., 2004). The α was calculated from the rate of the photosynthetic carbon reduction cycle and the fluorescence yield under non-photorespiratory (2% O_2_) conditions according to the method of Miyake and Yokota (2000).

### Estimation of the Flux of Alternative Electron Flow

The flux of electron transport through PSII (*J*_PSII_) was determined as described by Harley et al. (1992). The rate of oxygenation by Rubisco (*V*_o_) was measued according to von Caemmerer and Farquhar (1981). The rate of carboxylation by Rubisco (*V*_c_) was estimated following Miyake and Yokota (2000). Under atmospheric conditions, the electron fluxes in the two cycles can be expressed as *J*_c_ = 4 ×*V*_c_ and *J*_o_ = 4 ×*V*_o_, respectively. An alternative flux (*J*_a_) aroused by electrons that are not used by the carboxylation and/or oxygenation cycles on the total electron flux driven by PSII was estimated from *J*_PSII_ – *J*_c_ – *J*_o_ (Miyake and Yokota, 2000). O_2_-dependent *J*_a_ was determined from the difference between *J*_a_ (21% O_2_) and *J*_a_ (2% O_2_). Then, O_2_-independent *J*_a_ was measured from the difference between *J*_a_ and O_2_-dependent *J*_a_.

### Pigments Measurement

Pigments from xanthophyll cycle (V, violaxanthin; A, antheraxanthin; Z, zeaxanthin; L, lutein) were determined as described by Thayer and Björkman (1990). Briefly, fresh leaf tissue (0.3 g) was extracted with 80% acetone, filtered through a 0.45 μm membrane and quantified by HPLC (Waters 2695, USA). A Spherisorb C18 column (5 μm, 250 mm × 4 mm) was used with a flow rate of 1.5 mL min^-1^. Elution was conducted with acetonitrile/methanol (75:25, v/v) and methanol/ethyl acetate (70:30, v/v) as the A and B mobile phase. The mobile phase gradient was used as follows: start with 100% A for 7 min, increase to 100% B within 2 min, and then maintained for 23 min. The column was re-equilibrated with 100% A for 5 min prior to the next injection. The 10 μL sample was injected, and the pigments were detected by absorption measurements at 445 nm. The de-epoxidation state (DEPS) of xanthophyll cycle pool (VAZ) was calculated as (0.5A + Z)/(VAZ). Chlorophyll *a*+ *b* (Chl*a*+*b*) and total carotene (Car) were extracted in the same way and measured by spectrophotometry at 662, 645, and 470 nm, respectively (Sükran et al., 1998).

### Determination of ROS and Lipid Peroxidation

The producing rate of superoxide anion ($O_{2}^{•-}$) was measured by monitoring the nitrite formation from hydroxylamine in the presence of $O_{2}^{•-}$ (Zhou et al., 2004). Fresh leaf tissue (0.2 g) was homogenized with 2 mL of 65 mM phosphate buffer (pH 7.8) and centrifuged at 5000 *g* for 10 min. The incubation mixture contained 0.9 mL of 65 mM phosphate buffer (pH 7.8), 0.1 mL of 10 mM hydroxylammonium chloride and 1 mL of supernatant. After incubation at 25°C for 20 min, 17 mM sulfanilamide and 7 mM α-naphthylamine, were added to the incubation mixture and kept at 25°C for 20 min. Ethyl ether in the same volume was added and centrifuged at 1500 *g* for 5 min. The absorbance of the aqueous solution was read at 530 nm.

Hydrogen peroxide was determined by monitoring the absorbance of the titanium-peroxide complex (Zhou et al., 2004). Fresh leaf tissue (0.2 g) was homogenized with 5 mL of acetone and centrifuged at 3000 *g* for 10 min. The reactive mixture contained 0.1 mL of titanium reagent (50 μL of 20% titanium tetrachloride in concentrated HCl), 0.2 mL of ammonia and 1 mL of supernatant and centrifuged at 3000 *g* for 10 min. The resulting precipitate was washed five times with acetone and centrifuged at 10,000 *g* for 5 min. The precipitate was solubilized in 3 mL of 1 M H_2_SO_4_, and the absorbance was read at 410 nm.

Lipid peroxidation was estimated by analyzing MDA content according to the thiobarbituric acid (TBA) test. Fresh leaf tissue (0.2 g) was homogenized with 2 mL of 50 mM phosphate buffer (pH 7.8) and centrifuged at 12,000 *g* for 20 min. One milliliter of supernatant was mixed with 3 mL of 20% trichloroacetic acid (TCA) solution containing 2% TBA. The reactive mixture was heated in a water bath at 95°C for 30 min and centrifuged at 15,000 *g* for 10 min. The absorbance was read at 532 and 600 nm. The amount of MDA was calculated using an extinction coefficient of 155 mM^-1^ cm^-1^ (Heath and Packer, 1968).

### Purification of Cell Organelles

Organelles were isolated from leaves by differential and density-gradient centrifugation, according to the method of Mittova et al. (2000) and Song et al. (2009). Fresh leaf tissue (10 g) was chopped using a blender (HR-2826, Philips, China) with five volumes of medium per g FW in a medium containing 50 mM HEPES (pH 7.5), 5 mM γ-caproic acid, 0.3% BSA, 0.3 M sucrose, 10 mM NaCl, 5 mM Na-AsA, 10 mM β-mercaptoethanol, 2 mM EDTA and 1% PVP. The homogenates were filtered through four layers of gauze. The crude chloroplast fraction was sedimented by centrifugation at 1000 *g* for 5 min, purified by a Percoll discontinuous gradient (10, 40, 70, and 90%) and then recentrifuged at 4700 *g* for 15 min. An intact chloroplast layer was obtained from between 40 and 70% Percoll fraction. The harvested supernatant at 1000 *g* was recentrifuged at 12,000 *g* for 15 min, and then the pellets were collected. The collected pellets were resuspended in a medium containing 20 mM HEPES-KOH (pH 7.5), 330 mM sorbitol, 10 mM NaCl, and 2 mM EDTA. In this isolation procedure, the harvested supernatant at 12,000 *g* was considered to be the cytosol fraction. The collected pellets at 12,000 *g* were fractionated by a sucrose discontinuous gradient (25, 37, 45, and 57%) at 68,000 *g* for 3.5 h, and then an intact mitochondrial layer was obtained from between 37 and 45% sucrose fractions. The intactness of isolated chloroplasts and mitochondria were detected using the ferricyanide method and Cyt *c* method (Song et al., 2009), and found in the range of 80–90 and 75–85%, respectively. Interactive contamination (%) of the isolated organelles was calculated by dividing the activity of the respective marker enzyme (CCO, mitochondria; CAT, peroxisomes or chlorophyll content, chloroplasts) in the isolated organelle by its total activity (or amount) in the whole sucrose gradient. Although slight cross contaminations (in the range of 3–9%) of chloroplasts and cytosol by mitochondria, mitochondria and cytosol by chloroplasts, and chloroplasts, mitochondria and cytosol by peroxisomes were found, they were within an acceptable range (Mittova et al., 2000), suggesting the organelles were well-isolated. Finally, these isolated organelles were used for the following enzymes analyses.

### Antioxidative Enzymes Analyses

Superoxide dismutase (EC 1.15.1.1) activity was assayed as described by Giannopolitis and Ries (1977). The reactive mixture contained NBT solution, which consisted of 50 mM phosphate buffer (pH 7.8), 13 mM methionine, 63 μM NBT, 1.3 μM riboflavin, 0.1 mM EDTA and supernatant. One unit of SOD activity was defined as the amount of enzyme required to cause a 50% inhibition in the rate of *p*-nitro blue tetrazolium chloride reduction at 560 nm. Ascorbate peroxidase (EC 1.11.1.11) activity was estimated by monitoring the rate of AsA oxidation at 290 nm according to Nakano and Asada (1981). The reactive mixture contained 25 mM phosphate buffer with 0.1 mM EDTA (pH 7.0), 0.25 mM AsA, 1 mM H_2_O_2_ and supernatant. Glutathione reductase (EC 1.6.4.2) activity was determined by monitoring a decrease in absorbance at 340 nm caused by NADPH oxidation as described by Madamanchi and Alscher (1991). The reactive mixture contained 25 mM HEPES with 0.2 mM EDTA (pH 7.8), 0.12 mM NADPH, 0.5 mM GSSG and supernatant. Monodehydroascorbate reductase (EC 1.6.5.4) activity was measured by monitoring a decrease in absorbance at 340 nm due to NADH oxidation according to Arrigoni et al. (1981). The reactive mixture contained 25 mM HEPES with 0.2 mM EDTA (pH 7.8), 0.1 mM AsA, 0.5 unit AsA oxidase, 0.1 mM NADH and supernatant. Dehydroascorbate reductase (EC 1.8.5.1) activity was assayed by following the formation of AsA from DHA at 265 nm as described by Dalton et al. (1986). The reactive mixture contained 25 mM HEPES with 0.1 mM EDTA (pH 7.0), 0.4 mM DHA, 3.5 mM GSH and supernatant.

### Antioxidants Measurements

The reduced (AsA) and oxidized (DHA) ascorbate contents were determined according to the method of Law et al. (1983). Fresh leaf tissue (0.2 g) was extracted with 2 mL of 5% TCA and centrifuged at 15,000 *g* for 15 min. For total ascorbate (AsA + DHA) determination, 0.2 mL of supernatant was mixed with 0.5 mL of 150 mM phosphate buffer (5 mM EDTA, pH 7.4) and 0.1 mL of 10 mM DTT for 10 min and then with 0.1 mL of 0.5% *N*-ethylmaleimide. For AsA determination, 0.2 mL of supernatant was mixed with 0.5 mL of 150 mM phosphate buffer (5 mM EDTA, pH 7.4) and 0.2 mL of deionized H_2_O. Color was developed in both reactive mixtures adding 0.4 mL of 10% TCA, 0.4 mL of 44% orthophosphoric acid, 0.4 mL of 4% 2, 2′-bipyridyl and 0.2 mL of 3% FeCl_3_. Then, the mixtures were incubated at 40°C for 40 min and read the absorbance at 525 nm. DHA was calculated from the difference between AsA + DHA and AsA.

The reduced (GSH) and oxidized (GSSG) glutathione contents were measured following Zhou et al. (2007). Fresh leaf tissue (0.2 g) was extracted with 2 mL of 6% metaphosphoric acid and centrifuged at 12,000 *g* for 20 min. For total glutathione (GSH + GSSG) determination, the reactive mixture contained 1.6 mL of 100 mM phosphate buffer (pH 7.5), 0.1 mL of 0.6 mM DTNB, 0.1 mL of 0.2 mM NADPH, 0.1 mL of 50 U mL^-1^ GR and 0.1 mL of supernatant and quantified at 412 nm. GSSG was assayed according to the same method after removal of GSH by 0.03 mL of 2-vinylpyridine derivatizations at 25°C for 1 h. GSH was estimated by subtraction of GSSG from GSH + GSSG.

### Statistical Analysis

All variables within the same stage (drought and subsequent rewatering) were subjected to a one-way ANOVA due to the different treatment times (30 and 15 days), and the means of four replicates were compared by Duncan’s test at *P* < 0.05 level. Before ANOVA, the data were checked for normality and homogeneity of variances, and when needed, log-transformed to correct deviations from these assumptions. Linear regression was used to investigate the relationship among CO_2_ assimilation, lipid peroxidation, and energy partitioning. Statistical tests were performed using SAS 9.1 program (SAS Institute, Gary, NC, USA).

## Results

### Water Status of Leaves, Gas Exchange, and Chlorophyll Fluorescence

The LRWC significantly decreased in drought-stressed compared with WW plants (**Table 1**). Especially under SD condition, the plants showed more severe dehydration. After rewatering, LRWC of previously stressed plants returned to control level. Moreover, drought stress obviously decreased *P*_n_, *G*_s_, *F*_v_/*F*_m_, Φ_PSII_, and *q*_P_. Thereinto, *P*_n_ and *F*_v_/*F*_m_ respectively decreased by 40.1 and 10.0% in MD plants as well as by 61.3 and 16.3% in SD plants. On the contrary, NPQ increased by 38.5% in MD plants and by 93.6% in SD plants. It is important to report that *C*_i_ was only enhanced in MD plants. After rewatering, *P*_n_, *G*_s_, *C*_i_, Φ_PSII_, and *q*_P_ in SD plants restored to MD levels, which were still lower than those in WW plants. Differently, *F*_v_/*F*_m_ in SD plants was 3.9 and 7.4% lower than that in MD and WW plants, respectively, whereas NPQ in SD plants was 26.3 and 29.0% higher than that in MD and WW plants.

**Table 1:** Leaf water status, gas exchange and chlorophyll fluorescence parameters of *Fargesia rufa* plants under drought and rewatering.

Parameter	Drought phase			Rewatering phase		
	WW	MD	SD	WW	MD	SD
LRWC	90.93 ± 0.99 a	85.79 ± 1.49 b	80.84 ± 1.24 c	91.78 ± 1.28 a	89.33 ± 1.29 a	89.85 ± 1.65 a
*P*_n_	5.58 ± 0.22 a	3.34 ± 0.25 b	2.16 ± 0.15 c	5.06 ± 0.24 a	4.00 ± 0.20 b	3.77 ± 0.11 b
*G*_s_	138 ± 3 a	117 ± 9 b	57 ± 3 c	159 ± 3 a	102 ± 6 b	111 ± 3 b
*C*_i_	173 ± 6 b	219 ± 9 a	186 ± 9 b	235 ± 26 a	166 ± 9 b	209 ± 7 ab
*F*_v_/*F*_m_	0.80 ± 0.01 a	0.72 ± 0.01 b	0.67 ± 0.01 c	0.78 ± 0.01 a	0.75 ± 0.01 a	0.72 ± 0.02 b
Φ_PSII_	0.21 ± 0.01 a	0.19 ± 0.01 b	0.13 ± 0.01 c	0.19 ± 0.01 a	0.18 ± 0.00 ab	0.16 ± 0.01 b
*q*_P_	0.57 ± 0.01 a	0.45 ± 0.02 b	0.34 ± 0.02 c	0.49 ± 0.02 a	0.45 ± 0.01 ab	0.39 ± 0.01 b
NPQ	0.78 ± 0.01 c	1.08 ± 0.03 b	1.51 ± 0.03 a	0.95 ± 0.06 b	0.93 ± 0.04 b	1.20 ± 0.01 a

*Leaf relative water content (%), net CO_*2*_ assimilation rate (*P*_*n*_, μmol CO_*2*_ m^*–2*^ s^*–1*^), stomatal conductance (*G*_*s*_, mmol H_*2*_O m^*–2*^ s^*–1*^), intercellular CO_*2*_ concentration (*C*_*i*_, μmol CO_*2*_ mol^*–1*^), the intrinsic quantum efficiency of PSII photochemistry (*F*_*v*_/*F*_*m*_), the quantum yield of PSII reaction center photochemistry (Φ_*PSII*_), photochemical quenching (*q*_*P*_) and non-photochemical quenching (NPQ). Well-watered (WW), moderate drought (MD), and severe drought (SD). Means and SE of four replicates are shown. Different letters within the same stage indicate significant differences (*P* < 0.05) according to Duncan’s test*.

### Allocation of Energy Fluxes

**FIGURE 1:**
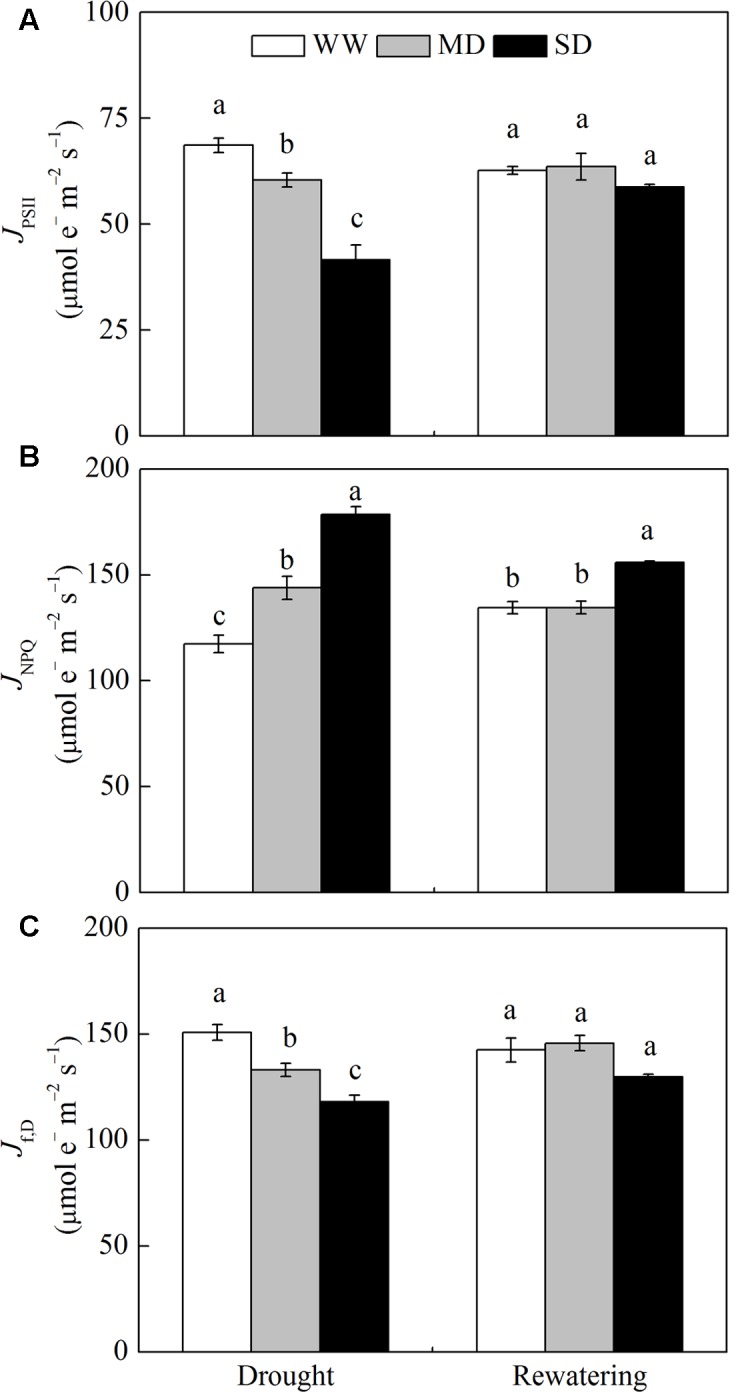
**Changes in energy dissipation flux *via* different pathways of *Fargesia rufa* plants under drought and rewatering. (A)** energy flux *via* linear electron transport in PSII (*J*_PSII_), **(B)** energy flux *via*ΔpH- and xanthophyll-mediated thermal dissipation (*J*_NPQ_), and **(C)** energy flux *via* fluorescence and constitutive thermal dissipation (*J*_f,D_). Well-watered (WW, open bars), moderate drought (MD, gray bars), and severe drought (SD, closed bars). Data are the means of four replicates with SE shown by vertical bars. Different letters within the same stage indicate significant differences (*P* < 0.05) according to Duncan’s test.

The *J*_PSII_ and *J*_f,D_ significantly decreased after drought stress, especially under SD condition (**Figures 1A,C**). In contrast, *J*_NPQ_ increased by 22.4% in MD plants and by 51.9% in SD plants compared with WW plants (**Figure 1B**). After rewatering, *J*_PSII_ and *J*_f,D_ in stressed plants fully recovered, whereas *J*_NPQ_ in SD plants was still 15.7% higher than that in WW plants (**Figure 1**).

**FIGURE 2:**
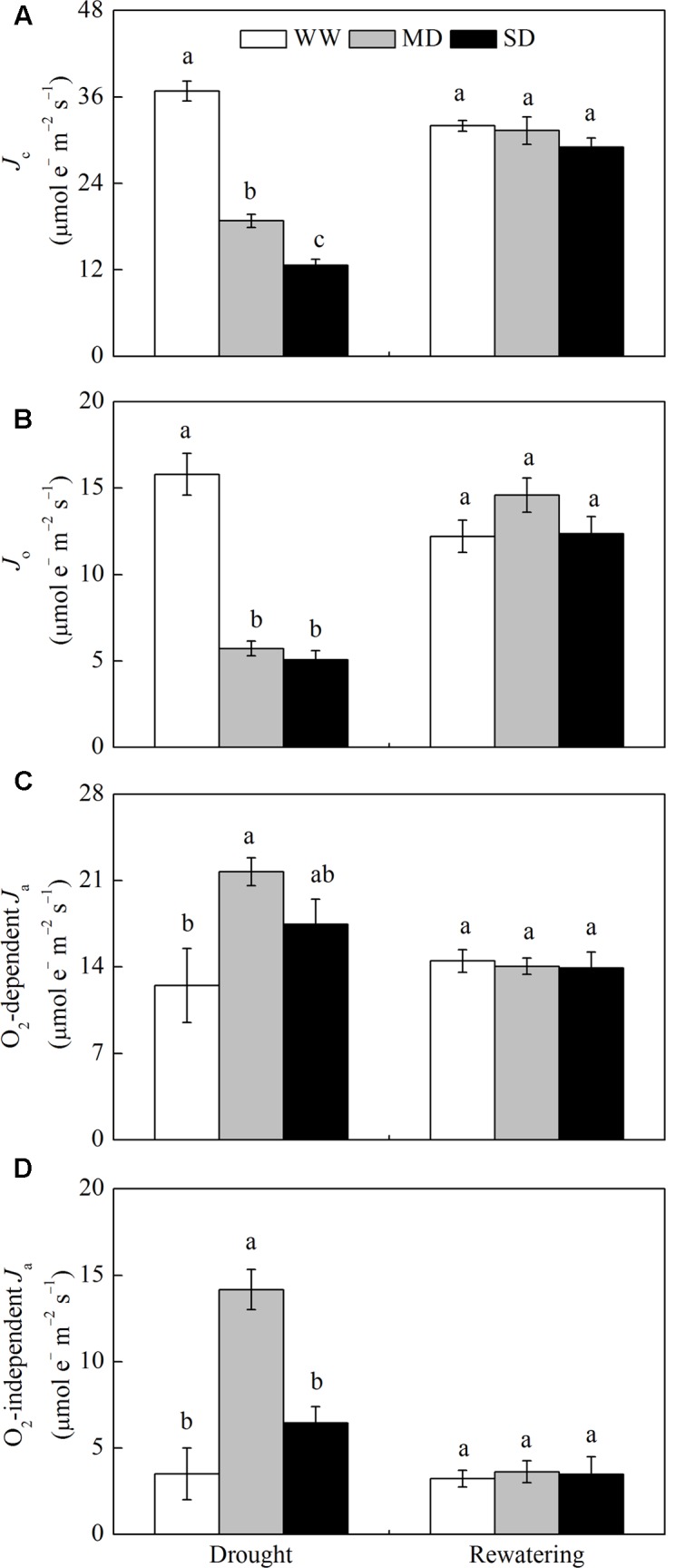
**Changes in the flux of electron transport *via* different proportions of linear electron transport in PSII (*J*_PSII_) of *F. rufa* plants under drought and rewatering. (A)** electron flux for photosynthetic carbon reduction *J*_c_, **(B)** electron flux for photorespiratory carbon oxidation *J*_o_, **(C)** O_2_-dependent alternative electron flux (O_2_-dependent *J*_a_), and **(D)** O_2_-independent alternative electron flux (O_2_-independent *J*_a_). Well-watered (WW, open bars), moderate drought (MD, gray bars), and severe drought (SD, closed bars). Data are the means of four replicates with SE shown by vertical bars. Different letters within the same stage indicate significant differences (*P* < 0.05) according to Duncan’s test.

For WW plants, *J*_c_, *J*_o_, O_2_-dependent *J*_a_ and O_2_-independent *J*_a_ accounted for approximately 53.6, 23.1, 18.2, and 5.1% of *J*_PSII_, respectively (**Figure 2**). Drought stress significantly decreased the proportion of *J*_c_ and *J*_o_, and increased the proportion of O_2_-dependent *J*_a_ and O_2_-independent *J*_a_. For example, in MD plants, *J*_c_, *J*_o_, O_2_-dependent *J*_a_ and O_2_-independent *J*_a_ accounted for approximately 31.2, 9.4, 35.9, and 23.5% of *J*_PSII_, respectively (**Figure 2**). Especially, the proportion of O_2_-independent *J*_a_ in MD plants was 8.0% higher than that in SD plants (**Figure 2D**). After rewatering, the proportions of these parameters in *J*_PSII_ in stressed plants returned to control levels (**Figure 2**).

### Pigments

As compared with WW plants, the content of xanthophyll cycle pigments (VAZ) obviously increased in MD plants, but decreased in SD plants (**Table 2**). Moreover, DEPS remained constant in MD plants although both V and Z contents increased. In comparison, DEPS increased dramatically by 22.3% in SD plants mainly due to significant decrease in V content. The higher L content was observed in drought-stressed than in WW plants. Also, the content of photosynthetic pigments (Chl*a*+*b* and Car) significantly decreased in SD plants. After rewatering, all these parameters were restored completely except higher Z content and DEPS in SD plants.

**Table 2:** Pigments content of *F. rufa* leaves under drought and rewatering.

Parameter	Drought phase			Rewatering phase		
	WW	MD	SD	WW	MD	SD
L	183.9 ± 2.7 c	193.5 ± 2.6 b	201.7 ± 3.0 a	190.0 ± 4.0 a	188.1 ± 4.8 a	193.9 ± 4.0 a
V	46.6 ± 0.7 b	52.6 ± 0.8 a	38.0 ± 0.6 c	47.0 ± 0.8 a	46.8 ± 1.2 a	44.3 ± 1.2 a
A	6.3 ± 0.3 b	7.1 ± 1.1 ab	7.9 ± 0.5 a	7.0 ± 0.3 a	6.2 ± 0.2 a	7.1 ± 0.6 a
Z	8.3 ± 0.2 b	9.3 ± 0.2 a	8.6 ± 0.1 b	6.4 ± 0.8 b	7.0 ± 0.8 ab	9.3 ± 0.9 a
VAZ	61.2 ± 0.6 b	68.9 ± 1.9 a	54.5 ± 0.9 c	60.4 ± 0.9 a	59.9 ± 0.3 a	60.7 ± 1.6 a
DEPS	18.8 ± 0.5 b	18.6 ± 0.3 b	23.0 ± 0.2 a	16.3 ± 1.4 b	16.8 ± 1.5 b	21.1 ± 0.5 a
Chl*a*+ *b*	2.61 ± 0.06 a	2.65 ± 0.15 a	1.82 ± 0.05 b	2.25 ± 0.03 a	2.23 ± 0.07 a	2.15 ± 0.11 a
Car	0.62 ± 0.01 a	0.63 ± 0.01 a	0.54 ± 0.01 b	0.60 ± 0.01 a	0.60 ± 0.01 a	0.58 ± 0.01 a

*Lutein (L), violaxanthin (V), antheraxanthin (A), zeaxanthin (Z) and xanthophyll cycle pool (VAZ) were expressed as mmol mol^*–1*^ Chl; the de-epoxidation state of VAZ-pool (DEPS, %); chlorophyll *a*+ *b* (Chl*a*+ *b*) and carotene (Car) were expressed as mg g^*–1*^ FW. Well-watered (WW), moderate drought (MD), and severe drought (SD). Means and SE of four replicates are shown. Different letters within the same stage indicate significant differences (*P* < 0.05) according to Duncan’s test*.

### ROS and Lipid Peroxidation

**FIGURE 3:**
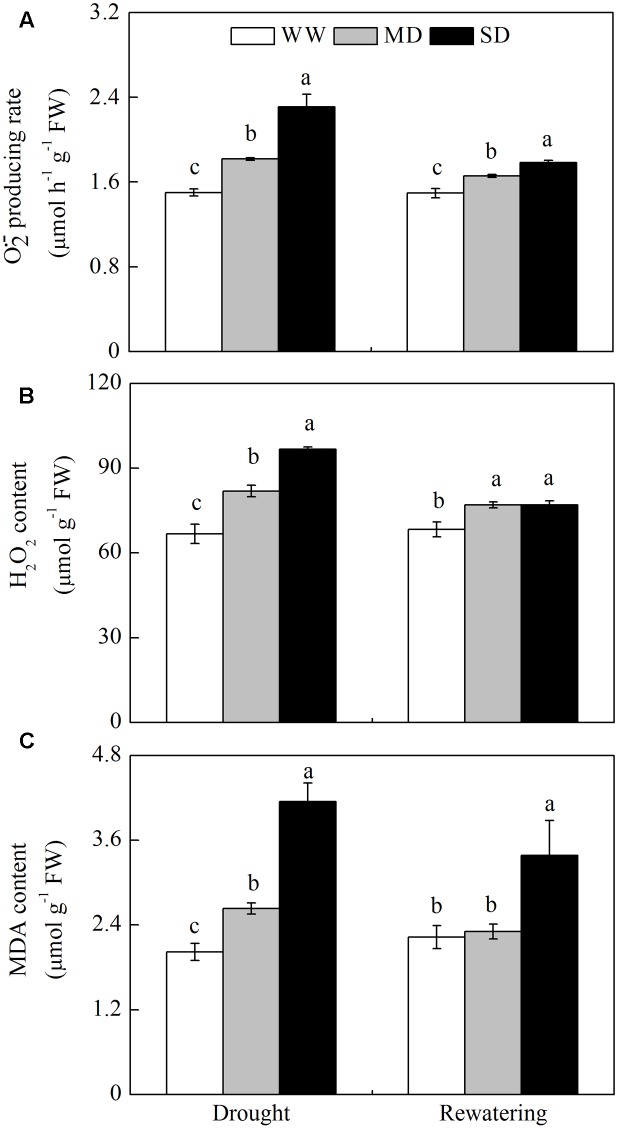
**Changes in reactive oxygen species (ROS) and lipid peroxidation of *F. rufa* plants under drought and rewatering. (A)** superoxide anion ($O_{2}^{•-}$) producing rate, **(B)** hydrogen peroxide (H_2_O_2_), and **(C)** lipid peroxidation (MDA content). Well-watered (WW, open bars), moderate drought (MD, gray bars), and severe drought (SD, closed bars). Data are the means of four replicates with SE shown by vertical bars. Different letters within the same stage indicate significant differences (*P* < 0.05) according to Duncan’s test.

The higher levels of $O_{2}^{•-}$ and H_2_O_2_ were detected in drought-stressed compared with WW plants, resulting in lipid peroxidation (given by MDA accumulation) (**Figure 3**). The MDA content increased by 30.2% in MD plants and by 105.4% in SD plants (**Figure 3C**). After rewatering, the levels of $O_{2}^{•-}$ and H_2_O_2_ in stressed plants were still higher than those in WW plants (**Figures 3A,B**). Comparatively, MDA content in MD plants fully recovered, but its content in SD plants was 51.6% greater than that in WW plants (**Figure 3C**).

### Activities of ROS-Scavenging Enzymes and Antioxidants

**FIGURE 4:**
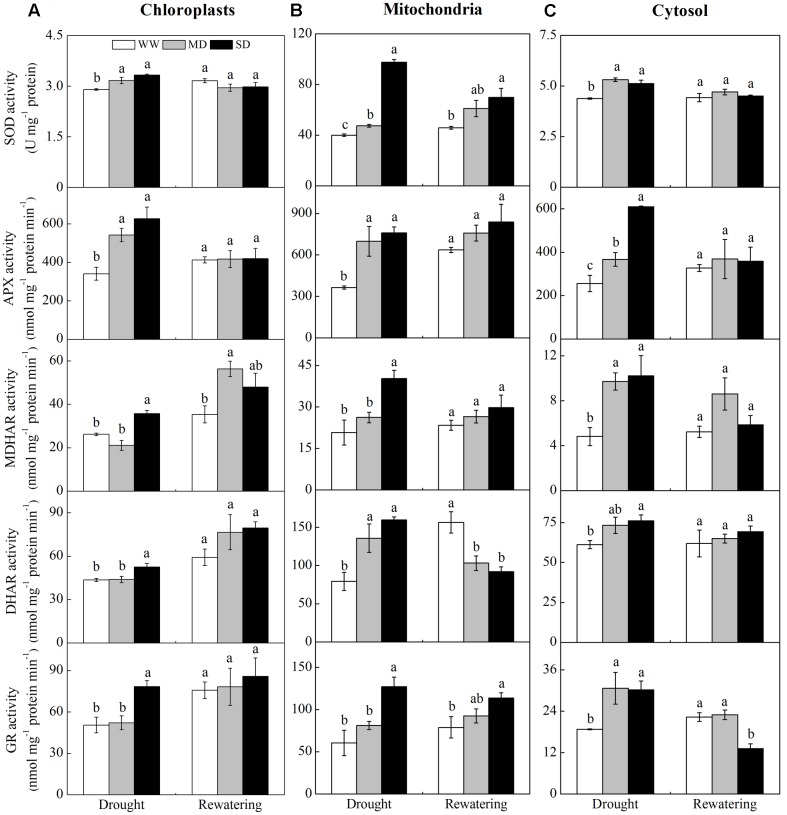
**Changes in the activities of antioxidative enzymes in chloroplasts (A)**, mitochondria **(B)**, and cytosol **(C)** from *F. rufa* leaves under drought and rewatering. Well-watered (WW, open bars), moderate drought (MD, gray bars), and severe drought (SD, closed bars). Data are the means of four replicates with SE shown by vertical bars. Different letters within the same stage indicate significant differences (*P* < 0.05) according to Duncan’s test.

Drought stress induced a general increase in the activities of ROS-scavenging enzymes localized in the chloroplasts, mitochondria, and cytosol. However, the activities of ROS scavenging enzymes in stressed plants returned to control levels after rewatering (**Figure 4**). In the chloroplasts, SOD and APX activities in drought-stressed plants were greater than those in WW plants (**Figure 4A**). However, MDHAR, DHAR, and GR activities only increased in SD plants. All enzymes activities fully recovered after rewatering, except that MDHAR activity was lower in MD than in WW plants. In the mitochondria, the activities of SOD, APX, and DHAR in stressed plants were greater than those in WW plants (**Figure 4B**), whereas MDHAR and GR activities increased only in SD plants. After rewatering, SOD and GR activities in stressed plants were still higher than those in WW plants, while DHAR activity was lower than that in WW plants. In the cytosol, all enzymes activities in stressed plants were higher than those in WW plants (**Figure 4C**). After rewatering, their activities completely recovered, except that GR activity was lower in SD than in WW plants. Moreover, DHAR activity was much higher than MDHAR activity in all cellular fractions of stressed plants. The mitochondria of stressed plants had higher SOD, DHAR, and GR activities than those observed in the chloroplasts and cytosol fractions (**Figure 4**).

The content of antioxidants (i.e., AsA + DHA, AsA, GSH + GSSG, and GSH) was not affected by drought stress, except AsA + DHA and AsA in SD plants, which respectively increased by 40.3 and 33.6% compared with WW plants (**Table 3**). However, drought stress significantly decreased the redox states of ascorbate and glutathione (i.e., AsA/DHA and GSH/GSSG). After rewatering, only AsA content and GSH/GSSG in SD plants were still higher than those in WW plants.

**Table 3:** Antioxidants content of *F. rufa* leaves under drought and rewatering.

Parameter	Drought phase			Rewatering phase		
	WW	MD	SD	WW	MD	SD
AsA + DHA	3.00 ± 0.03 b	3.40 ± 0.05 b	4.21 ± 0.24 a	3.24 ± 0.16 a	3.27 ± 0.07 a	3.46 ± 0.14 a
AsA	2.74 ± 0.02 b	3.03 ± 0.04 b	3.66 ± 0.23 a	3.06 ± 0.03 b	3.01 ± 0.05 b	3.24 ± 0.04 a
AsA/DHA	10.86 ± 0.49 a	8.17 ± 0.66 b	6.64 ± 0.39 b	10.42 ± 0.98 a	12.15 ± 0.72 a	10.78 ± 0.85 a
GSH + GSSG	0.73 ± 0.04 ab	0.62 ± 0.06 b	0.79 ± 0.01 a	0.66 ± 0.03 a	0.59 ± 0.03 a	0.60 ± 0.03 a
GSH	0.55 ± 0.04 a	0.46 ± 0.05 a	0.55 ± 0.01 a	0.48 ± 0.02 a	0.44 ± 0.03 a	0.45 ± 0.02 a
GSH/GSSG	3.01 ± 0.20 a	2.92 ± 0.22 ab	2.35 ± 0.05 b	2.54 ± 0.21 b	2.84 ± 0.14 ab	3.21 ± 0.19 a

*The reduced (AsA) and the oxidized (DHA) form of ascorbate as well as the reduced (GSH) and the oxidized (GSSG) form of glutathione were expressed as μmol g^*–1*^ FW. Well-watered (WW), moderate drought (MD), and severe drought (SD). Means and SE of four replicates are shown. Different letters within the same stage indicate significant differences (*P* < 0.05) according to Duncan’s test*.

### Relationship among CO_2_ Assimilation, Lipid Peroxidation, and Energy Partitioning

**FIGURE 5:**
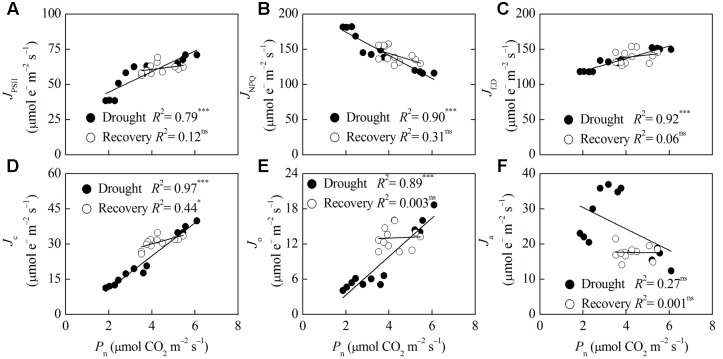
**Relationship between net CO_2_ assimilation (*P*_n_) and different energy partitioning processes in *F. rufa* leaves under drought and rewatering**. The regression lines are: **(A)** *y* = 7.31x + 29.90, **(B)** *y* = –16.51x + 207.48, **(C)** *y* = 8.87x + 101.29, **(D)** *y* = 7.06x – 3.30, **(E)** *y* = 3.29x – 3.29, **(F)** *y* = –3.04x + 36.49 (for drought); and **(A)** *y* = 1.92x + 52.98, **(B)** *y* = –9.36x + 181.61, **(C)** *y* = 3.47x + 124.58, **(D)** *y* = 2.57x + 19.78, **(E)** *y* = 0.15x + 12.41, **(F)** *y* = –0.07x + 17.90 (for rewatering). Data are measured values of four replicates per treatment at the same stage (error bars are omitted for clarity). The solid lines represent the best-fit linear regressions: ^∗^*P* < 0.05; ^∗∗∗^*P* < 0.001; ns, not significant.

**FIGURE 6:**
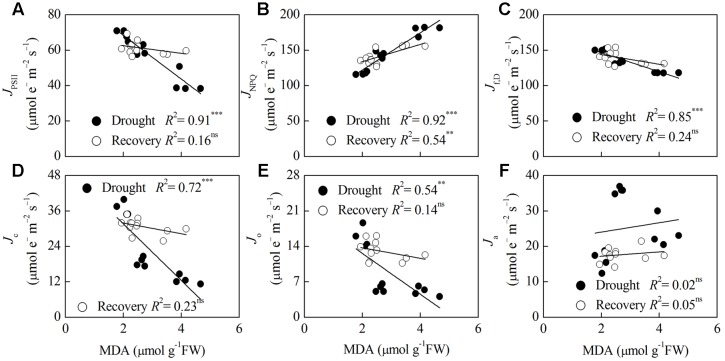
**Relationship between lipid peroxidation (MDA) and different energy partitioning processes in *F. rufa* leaves under drought and rewatering**. The regression lines are: **(A)** *y* = –12.31x + 92.98, **(B)** *y* = 26.33x + 69.35, **(C)** *y* = –13.46x + 173.50, **(D)** *y* = –9.60x + 50.89, **(E)** *y* = –4.03x + 20.69, **(F)** *y* = 1.32x + 21.40 (for drought); and **(A)** *y* = –2.17x + 66.90, **(B)** *y* = 12.19x + 109.43, **(C)** *y* = –6.84x + 157.44, **(D)** *y* = –1.82x + 35.60, **(E)** *y* = –1.04x + 15.79, **(F)** *y* = 0.61x + 16.00 (for rewatering). Data are measured values of four replicates per treatment at the same stage (error bars are omitted for clarity). The solid lines represent the best-fit linear regressions: ^∗∗^*P* < 0.01; ^∗∗∗^*P* < 0.001; ns, not significant.

Correlation analysis of *P*_n_, MDA and energy partitioning (**Figures 5**, **6**) showed that *P*_n_ was positively correlated with *J*_PSII_, *J*_f,D_, *J*_c_, and *J*_o_, and was negatively correlated with *J*_NPQ_ after drought stress (*P* < 0.001) (**Figures 5A–E**). *P*_n_ only had a positive correlation with *J*_c_ after rewatering (*P* < 0.05) (**Figure 5D**). Contrastingly, MDA was negatively correlated with *J*_PSII_, *J*_f,D_, *J*_c_ and *J*_o_, and was positively correlated with *J*_NPQ_ after drought stress (*P* < 0.01) (**Figures 6A–E**). MDA only had a positive correlation with *J*_NPQ_ after rewatering (*P* < 0.01) (**Figure 6B**). Regardless of drought or rewatering phase, there were no significant correlations between *P*_n_ and *J*_a_, as well as MDA and *J*_a_ (**Figures 5F**, **6F**).

## Discussion

### CO_2_ Assimilation under Drought and Rewatering

Photosynthesis is one of the most sensitive physiological processes to stressful environments such as drought and high temperature, and it is severely affected in all its phases by such stresses. Furthermore, PSII is considered to play an important role in the response of photosynthesis to environmental interferences (Tian et al., 2013). Depression of photosynthetic capacity is therefore found one of the key indicators of the decrease in PSII activity (Kong et al., 2015). Our study showed that the reduction of *P*_n_ in *F. rufa* plants under drought was primarily attributed to impairment of photosynthetic apparatus, especially PSII, as indicated by the decreases in *F*_v_/*F*_m_ and Φ_PSII_ (a measurement of the functional status of PSII) with higher *C*_i_ (**Table 1**). Moreover, the decrease of *P*_n_ under drought might be partly responsible for stomatal closure, which was demonstrated by a decline in *G*_s_ with decreasing LRWC. The photosynthetic capacity of C_3_ plants cannot be restored when *G*_s_ drops below 50–100 mmol H_2_O m^-2^ s^-1^ (Flexas et al., 2004, 2006), and when *P*_n_ is reduced by over 80% (Cornic and Fresneau, 2002). With full recovery of LRWC, the reduced *P*_n_ in MD plants (<80%) improved substantially due to complete recovery of PSII activity and partial recovery of *G*_s_ after rewatering (**Table 1**). Thus, stomatal closure may account mainly for the delayed recovery of *P*_n_ in MD plants. In contrast, SD plants displayed the only partial recovery of photochemistry suggesting a persisting metabolic impairment, at least partially inactive PSII units. However, Liu et al. (2014) found complete recovery of *P*_n_ in *Fargesia denudata* plants from SD condition. Thus, different dwarf bamboo species have certain differences in the photochemical response to drought stress.

### Different Photoprotective Pathways under Drought and Rewatering

In our study, photosynthetic pigments in *F. rufa* leaves maintained constant levels under MD, but declined under SD; and recovered to normal levels after rewatering (**Table 2**). This corroborated previous studies on *Arbutus unedo* (Munné-Bosch and Peñuelas, 2004) and *Jatropha curcas* (Silva et al., 2012). The adjustment of photosynthetic pigments will contribute to some degree of photoprotection under SD condition. Niyogi (1999) speculated that Chl and Car change to balance the absorption and utilization of light energy when plants are subjected to drought. Moreover, photosynthetic pigments loss may be a regulatory mechanism geared to reducing the amount of energy absorbed by leaves during drought; thus, decreasing energetic pressure at the PSII level (Munné-Bosch and Alegre, 2000). The effect of drought on photosynthetic pigments may vary with plant adaptation to habitat (Galmés et al., 2007a).

Drought decreases a plant’s capacity to assimilate CO_2_ thereby decreasing the demand for reducing equivalents (Wujeska et al., 2013), which creates an imbalance between the absorption and utilization of radiant energy that eventually results in excess excitation energy (Flexas and Medrano, 2002). Thermal dissipation (NPQ) involving the xanthophyll and presumably lutein cycles is one of the efficient strategies for the safe removal of excess energy. The capacity of thermal dissipation of *F. rufa* plants strongly increased while *P*_n_ was suppressed, especially under SD condition, as indicated by higher NPQ and DEPS (e.g., their correlation *r* = 0.85, *P* < 0.01) as well as more abundant L (**Tables 1**, **2**). This can also be confirmed by increased *J*_NPQ_ (nearly half of the absorbed energy, 43–53%) in stressed plants concomitantly with decreases in *J*_PSII_ and *J*_f,D_ (**Figure 1**). In this case, decrease of *F*_v_/*F*_m_ eventully occurred, which may be a consequence of drought-induced *P*_n_ decline rather than its cause because *J*_PSII_ (Galmés et al., 2007a). After rewatering, thermal dissipation was still operated largely under SD condition though *F*_v_/*F*_m_ did not recover. This redistribution of absorbed energy under drought conditions helps protect the photosynthetic apparatus from photoinhibition and accelerate its recovery once drought is relieved.

In addition, the contents of xanthophyll cycle pigments (VAZ) also contribute to NPQ. Previous studies showed that the VAZ increases or remains constant in parallel with DEPS in different plant species subjected to varying drought intensities (Munné-Bosch and Peñuelas, 2004; Peñuelas et al., 2004; Gallé et al., 2007; Galmés et al., 2007a). In our study, the accumulation of VAZ-pool in *F. rufa* plants was found under MD condition (**Table 2**), as can facilitate NPQ induction and thus play a photoprotective function in this case. Interestingly, under SD condition and subsequent rewatering, we observed a decreased or constant VAZ-pool accompanied by an increase in DEPS that was attributable to activation of violaxanthin de-epoxidase (VDE) by the acidification of the thylakoid lumen. This is consistent with the experimental result observed in *Pistacia lentiscus* plants (Munné-Bosch and Peñuelas, 2003). Thus, the role VAZ-pool in NPQ induction may have a certain relationship with (rely on) the intensity of stress.

The reduction in *J*_PSII_ in *F. rufa* plants was lower than the decline in the capacity of CO_2_ assimilation (*P*_n_ and *J*_c_) under drought condition, suggesting an alternative sink such as the water–water cycle (Miyake, 2010). We observed an increase in O_2_-dependent *J*_a_ that is driven by the water–water cycle, especially under MD condition (**Figure 2C**), and also O_2_-dependent *J*_a_/*J*_PSII_ was greater than the values previously reported for other C_3_ plants subjected to drought (Biehler and Fock, 1996; Lovelock and Winter, 1996). These results suggest that the water–water cycle can effectively operate by *F. rufa* plants to dissipate excess excitation energy, although its function is limited under SD condition. In contrast, Driever and Baker (2011) demonstrated that the water–water cycle is not a major alternative electron sink for dissipation of excess excitation energy when CO_2_ assimilation is restricted. Furthermore, A higher proportion of O_2_-independent *J*_a_ in stressed plants, especially under MD, may be used as a candidate for nitrate assimilation or a cyclic flow of electron within PSII (Miyake and Yokota, 2000), but the specific nature of this alternative electron sink is not known.

Enhanced photorespiration also serves as a safety valve to dissipate excess excitation energy during mild to moderate drought when *C*_i_ and *G*_s_ (>150 mmol H_2_O m^-2^ s^-1^) rather than Rubisco activity limit photosynthetic capacity (Guan et al., 2004; Galmés et al., 2007a; Silva et al., 2010, 2015; Abogadallah, 2011). However, lower *J*_o_ value and *J*_o_/*J*_PSII_ in our study indicated that photorespiration is not a major energy dissipation strategy in stressed *F. rufa* plants, as observed results in some Mediterranean plants (Nogués and Alegre, 2002). Hence, whether photorespiration plays a protective role may depend on differential inhibition of photosynthesis under stress conditions.

### ROS Metabolism under Drought and Rewatering

Restriction in CO_2_ assimilation under drought inevitably increases ROS in different processes of electron transport. Within plant cell organelles, the chloroplasts and mitochondria are the two main sites of ROS generation, while the cytosol acts as a sink for H_2_O_2_ leaked from other cellular compartments (Møller et al., 2007; Noctor et al., 2014; Kasote et al., 2015). The oxidative damage occurred in stressed *F. rufa* plants, especially under SD, as shown by obvious ROS ($O_{2}^{•-}$ and H_2_O_2_) accumulation and lipid peroxidation (MDA) (**Figure 3**). Meanwhile, the activities of ROS-scavenging enzymes in isolated organelles were activated substantially (**Figure 4**). Wherein, the activities of SOD and the enzymes involved in AsA-GSH cycle (APX, DDHAR, MDHAR, and GR) in mitochondria exhibited the most significant increases, which partly decrease the ROS accumulation. Thus, the differential responses of enzymes in different organelles of *F. rufa* plants may display an novel solution for removing harmful ROS.

Furthermore, AsA and GSH can also detoxify ROS (Gallé et al., 2007; Silva et al., 2010), and their levels increased in reponse to drought-induced ROS accumulation (Silva et al., 2012, 2015). In our study, DHAR activity was much higher than MDHAR activity in isolated organelles suggesting that AsA is recycled mainly via GSH oxidation, and this is more apparent in mitochondria than in chloroplasts and cytosol. Accordingly, a gradual increase in AsA content was observed after drought although GSH remained constant, resulting in lower ratios of AsA/DHA and GSH/GSSG (**Table 3**). This suggests that AsA in *F. rufa* plants plays a certain role in decreasing oxidative damage. After rewatering, SD plants still kept high levels of AsA reiterating its role in preventing oxidative damage.

### Relationship among CO_2_ Assimilation, Lipid Peroxidation, and Energy Partitioning

The capacity of CO_2_ assimilation or lipid peroxidation is closely related to absorption and allocation of light energy in leaves (Zhou et al., 2004, 2007). Therefore, we conducted correlation analysis between *P*_n_ or MDA and different energy partitioning processes in *F. rufa* plants at different phases. Our study showed highly positive correlations between *P*_n_ and *J*_c_ or *J*_o_ at the drought and rewatering phase, but there were no significant correlations between *P*_n_ and *J*_a_ (**Figures 5**, **6**). This result suggests that a drought-induced decrease in *J*_c_ was mostly compensated by *J*_a_ rather than by *J*_o_, resulting in improvement of *P*_n_ upon rewatering. Hence, *J*_a_ can be used as a sink for excess electrons in stressed *F. rufa* plants. When *P*_n_ was restricted at the drought stage, the ROS levels and lipid peroxidation did not clearly indicate decreased photorespiration as evident from negative correlation between MDA and *J*_o_. However, this is not surprising as oxidative stress is determined not only by photorespiration but also by other electron transport chains and overall changes in the redox status (Foyer and Noctor, 2005; Noctor et al., 2014). Moreover, highly correlations between *P*_n_ and *J*_NPQ_ as well as MDA and *J*_NPQ_ at the drought and rewatering phase showed that thermal dissipation could efficiently regulates energy utilization for CO_2_ assimilation to alleviate oxidative damage.

## Conclusion

To our knowledge, this is the first to use a systematic approach for evaluating the environmental stress on photoprotective pathways in a bamboo species, utimately aiming to identify how it resists drought stress and recovers once drought is relieved. The present study showed that drought down-regulates the capacity of CO_2_ assimilation in *F*. *rufa* plants and causes ROS-induced lipid peroxidation. However, *F*. *rufa* plants employ a network of photoprotective pathways including the water–water cycle (especially under moderate drought) as well as thermal dissipation and antioxidative defense capacity at organelle levels (especially under severe drought), to preserve the potential functionality of photosynthetic apparatus under varying intensities of drought, leading to the rapid recovery of photosynthetic performance after rewatering. Thus, *F*. *rufa* is capable of resisting and surviving drought environment.

## Author Contributions

CL, YW, and KP designed the practical part of the study, analyzed the data, and drafted the manuscript. JL, QW, and YJ carried out the physiologic studies and helped to revise the manuscript. AT contributed reagents/materials/analysis tools.

## Conflict of Interest Statement

The authors declare that the research was conducted in the absence of any commercial or financial relationships that could be construed as a potential conflict of interest.

## Abbreviations

A: antheraxanthin
APX: ascorbate peroxidase
AsA: reduced ascorbate
Car: carotene
*C*_i_: intercellular CO_2_ concentration
DEPS: de-epoxidation state of xanthophyll cycle pool
DHA: oxidized ascorbate
DHAR: dehydroascorbate reductase
*F*_v_/*F*_m_: intrinsic quantum efficiency of PSII photochemistry
GR: glutathione reductase
*G*_s_: stomatal conductance
GSH: reduced glutathione
GSSG: oxidized glutathione
H_2_O_2_: hydrogen peroxide
*J*_a_: alternative electron flux
*J*_c_: electron flux for photosynthetic carbon reduction
*J*_o_: electron flux for photorespiratory carbon oxidation
*J*_f,D_: energy flux *via* fluorescence and constitutive thermal dissipation
*J*_NPQ_: energy flux *via*ΔpH- and xanthophyll-mediated thermal dissipation
*J*_PSII_: energy flux *via* linear electron transport in PSII
L: lutein
LRWC: leaf relative water content
MDA: malondialdehyde
MDHAR: monodehydroascorbate reductase
NPQ: non-photochemical quenching
O_2_-dependent *J*_a_: O_2_-dependent alternative electron flux
O_2_-independent *J*_a_: O_2_-independent alternative electron flux
$O_{2}^{•-}$: superoxide anion
*P*_n_: net CO_2_ assimilation rate
*q*_p_: photochemical quenching
SOD: superoxide dismutase
V: violaxanthin
Z: zeaxanthin
Φ_PSII_: quantum yield of PSII reaction center photochemistry.
